# Cost-minimisation analysis of pegylated liposomal doxorubicin hydrochloride *versus* topotecan in the treatment of patients with recurrent epithelial ovarian cancer in Spain

**DOI:** 10.1038/sj.bjc.6601228

**Published:** 2003-09-09

**Authors:** B Ojeda, L M de Sande, A Casado, P Merino, M A Casado

**Affiliations:** 1 Department of Medical Oncology, Hospital de la Santa Cruz y San Pablo, Avgda. Sant Antoni María Claret 167, 08025 Barcelona, Spain; 2 Department of Medical Oncology, Hospital Virgen Blanca, Altos de Nava s/n, 24008 León, Spain; 3 Department of Medical Oncology, Hospital Clínico San Carlos, Doctor Martín Lagos s/n, 28040 Madrid, Spain; 4 Schering-Plough, Paseo de la Castellana 143, 28046 Madrid, Spain

**Keywords:** cost-minimisation, pegylated liposomal doxorubicin hydrochloride, topotecan, ovarian cancer, ovarian neoplasm

## Abstract

The study consisted of a cost-minimisation analysis since the findings from a multicentre randomised phase III trial showed that pegylated liposomal doxorubicin hydrochloride was at least as efficacious as topotecan. An economic model from the Spanish hospitals perspective was constructed to compare the costs derived from the treatment using both drugs in patients with recurrent epithelial ovarian cancer who failed a first-line platinum-containing regimen. The cost evaluation included direct medical costs: drug, drug administration and costs of managing adverse events. Estimation of resources used in managing adverse events was made retrospectively through an expert panel. Results obtained per patient were: cost of drug and administration, 8647.70 euros for pegylated liposomal doxorubicin hydrochloride and 8519.94 euros for topotecan, while cost of managing adverse events was 967.02 euros in the pegylated liposomal doxorubicin hydrochloride arm and 3304.75 euros for topotecan. The total cost per patient was therefore estimated to be 9614.72 euros for pegylated liposomal doxorubicin hydrochloride and 11 824.69 euros for topotecan, showing that pegylated liposomal doxorubicin hydrochloride produces a cost saving of 2209.97 euros per patient in comparison to topotecan. Sensitivity analyses verified the robustness of the results. These findings suggest that pegylated liposomal doxorubicin hydrochloride is an efficient therapy and can be used as a cost-saving option for treatment of patients with recurrent epithelial ovarian cancer who have failed a first-line platinum-containing regimen.

Ovarian cancer is the sixth most common form of cancer worldwide and is the leading cause of cancer deaths of the gynaecological tumours (Landis *et al*, 1998). In Spain, ovarian cancer has an annual incidence of 7.9 per 100 000 women, and an annual death rate of 3.7 per 100 000 women (Borrás *et al*, 1997). Due to the often asymptomatic nature of the early stages of disease, many cases of ovarian cancer have a very poor diagnosis and are not detected until the advanced stages, which suggest that a low survival rate since 5-year survival rates for women in advanced stages of the disease are only around 30% (Gore, 1999, pp 76–90). In advanced stage disease, standard care consists of surgical debulking followed by platinum-based therapy. Recent studies have helped to form the opinion that these advanced stage patients may be treated with paclitaxel and platinum therapy, due to increased response and survival rates with the combination (McGuire *et al*, 1996; Du Bois *et al*, 1999; Ozols *et al*, 1999; Piccart *et al*, 2000; Neijt *et al*, 2000). In spite of these improvements, 50–75% of patients with advanced disease will ultimately relapse (Hager *et al*, 2001). Some of these patients are candidates for retreatment with platinum regimens, but patients who do not respond or who relapse are much less likely to respond to subsequent chemotherapy regimens. There is a need for other effective treatments for platinum-resistant tumours.

Substantial clinical activity with pegylated liposomal doxorubicin hydrochloride (PLD; Caelyx/Doxil®) has been shown in patients refractory to paclitaxel and platinum-based chemotherapy (Gordon *et al*, 2001). Pegylated liposomal doxorubicin hydrochloride is a pegylated liposomal formulation of doxorubicin hydrochloride, which results in an altered kinetic profile, extending the half-life to 74 h. It is thought that the pegylated liposomal formulation may improve specificity of delivery to tumours, decreasing absorption by normal tissues, and may decrease many of the dose-limiting side effects of doxorubicin (Goram *et al*, 2001).

Topotecan (T) is a topoisomerase I inhibitor approved in Europe for the treatment of metastatic ovarian cancer after failure of first-line or for subsequent retreatment. Topotecan produces DNA damage in the presence of the nuclear enzyme topoisomerase I, which relieves the strain in DNA supercoils during replication and translation, and causes cell death by generating double-stranded DNA breaks (Kollmannsberger *et al*, 1999).

Recent guidance from NICE in the UK recommends that either PLD or T should be considered as options for the second-line (or subsequent) treatment of women with advanced ovarian cancer where the disease is initially resistant or refractory to first-line platinum-based combination therapy or has become resistant after successive courses of platinum-based combination therapy (National Institute for Clinical Excellence, 2001,^2002^).

A cost-minimisation analysis of PLD and T has been reported in a previous study in the USA and the UK (Smith *et al*, 2002). The objective of this analysis is to determine the most cost-efficient therapy in the Spanish setting, since the clinical findings from a multicentre randomised trial showed that PLD was at least as efficacious as T (Gordon *et al*, 2001). An economic analysis has been performed in the form of a cost-minimisation analysis, in order to estimate and compare the costs associated with the two alternatives, PLD and T in Spain.

## MATERIALS AND METHODS

### Analysis design

A cost-minimisation analysis is a type of pharmacoeconomic analysis in which costs of two or more alternative therapies are compared, and has as its central assumption that the clinical outcomes of those therapies are equivalent. The goal is to find the least expensive way of achieving the specific outcomes (Robinson, 1993; Basskin, 1998; Briggs and O'Brien, 2001).

This analysis was performed according to the findings of a randomised phase III trial, comparing PLD *vs* T in patients with recurrent epithelial ovarian cancer who failed a first-line platinum-containing regimen (Gordon *et al*, 2001). Since the findings from the clinical trial showed that PLD had overall comparable efficacy with T, the pharmacoeconomic analysis has been performed as a cost-minimisation analysis, wherein the costs of PLD and T are compared.

Our analysis consisted in a pharmacoeconomic model that allows simulations of complex clinical management to be made, and which uses estimations taken from published data of efficacy, tolerability and costs of the competing treatments. This model permitted us to estimate the expected economic consequences of a whole treatment process with PLD or T (Smith *et al*, 2002).

### Clinical data

The randomised phase III trial explained above was a multicentre study with sites both in Europe and North America. A total of 474 patients were treated (239 PLD and 235 T). All patients had failed first-line chemotherapy with a platinum-based regimen and comprised the intent-to-treat population. Patients were stratified prospectively for platinum sensitivity and bulky disease, and could have received no more than one prior platinum-based regimen. The study drug regimens consisted of either a 1 h intravenous infusion of PLD 50 mg m^−2^ every 28 days or T 1.5 mg m^−2^ day^−1^ as a 30 min infusion for five consecutive days every 21 days (Gordon *et al*, 2001; Smith *et al*, 2002).

The results obtained suggest comparable efficacy between the two drugs, PLD and T: the overall progression-free survival rates were similar between the two arms (113 days in the PLD arm and 119 in the T (*P*=0.095)); the overall response rates for PLD and T were 19.7 and 17.0%, respectively (*P*=0.390); median overall survival times were 60 weeks for the subgroup of PLD and 56.7 weeks for T (*P*=0.341). Data analysed in platinum-sensitive patients demonstrated a statistically significant benefit with PLD for progression-free survival (*P*=0.037), with medians of 28.9 weeks for PLD *vs* 23.3 weeks for T. For overall survival, PLD was significantly superior to T (*P*=0.008), with a median of 108 *vs* 71.1 weeks. The platinum-refractory subgroup demonstrated a nonstatistically significant survival trend in favour of T (0.455). Severe a haematologic toxicity was more common with T (Gordon *et al*, 2001).

### Resource estimation perspective

Cost estimation for a pathology treated with any drug is made by identification of the resources used in the process, measuring and quantifying these resources and assigning a standard price to each of these resources (in monetary units).

Usually, in this type of pharmacoeconomic analysis, only direct medical costs are included, without taking into account indirect and intangibles costs.

Three main categories of costs, derived from routine clinical practice in the Spanish hospitals, were considered in the cost-minimisation analysis:

- costs of the study drug;
- costs of drug administration (e.g. cost of outpatient visits, infusions);
- costs of managing adverse events (e.g. cost of additional medication, any associated hospitalisation).

To estimate the costs derived from each arm of the trial, the total amount of study drug per patient, used during the clinical trial, was calculated by multiplying each dose (mg m^−2^) administered by the patient's surface area and summing overall doses administered. The result was then multiplied by the cost of the study drug. The cost for outpatient consultations (at the beginning of each treatment cycle) and outpatient visits (for administration of each dose) were also added. In each administration of T, the use of antiemetic drugs (granisetron and tropisetron, in 66.7 and 33.3% of patients, respectively, according to expert opinion) was also required. Table 1

**Table 1:** Amounts of study drug and number of cycles (Gordon *et al*, 2001; Smith *et al*, 2002)

	**Total mg drug used**	**Mg per patient**	**Total cycles**	**Cycles per patient**	**Total doses**
**PLD**					
(*n*=239)	94 447	395.18	1164	4.87	1164

**Topotecan**					
(*n*=235)	15 653	66.60	1349	5.74	6673

PLD=pegylated liposomal doxorubicin hydrochloride.

shows the amounts of study drug and cycles (total and per patient) used to make these estimations (Gordon *et al*, 2001; Smith *et al*, 2002).

The resources used to manage every adverse event were measured for each drug, PLD and T. First, frequency of adverse events by type and severity level for both drugs was analysed, as shown in Table 2

**Table 2:** Adverse event frequency by type and severity level in the two treatment arms (Gordon *et al*, 2001; Smith *et al*, 2002)

**Adverse event type**	**Grade**	**PLD *n* (%)**	**Topotecan *n* (%)**
Anemia	Total	319 (100)	985 (100)
	Grade I	199 (62)	363 (37)
	Grade II	101 (32)	476 (48)
	Grade III	18 (6)	134 (14)
	Grade IV	1 (0)	12 (1)

Thrombocytopenia	Total	71 (100)	944 (100)
	Grade I	54 (76)	434 (46)
	Grade II	14 (20)	272 (29)
	Grade III	3 (4)	175 (19)
	Grade IV	0 (0)	63 (7)

Neutropenia	Total	311 (100)	1438 (100)
	Grade I	153 (48)	296 (20)
	Grade II	105 (35)	378 (26)
	Grade III	42 (14)	427 (30)
	Grade IV	11 (3)	337 (24)

Sepsis	Total	4 (100)	17 (100)
	Grade I	0 (0)	0 (0)
	Grade II	1 (25)	5 (35)
	Grade III	3 (75)	3 (15)
	Grade IV	0 (0)	9 (50)

Fever	Total	69 (100)	65 (100)
	Grade I	36 (55)	31 (48)
	Grade II	26 (42)	20 (31)
	Grade III	2 (3)	8 (12)
	Grade IV	0 (0)	5 (9)

Stomatitis/Pharyngitis	Total	378 (100)	130 (100)
	Grade I	202 (53)	91 (70)
	Grade II	144 (38)	37 (28)
	Grade III	31 (8)	2 (2)
	Grade IV	1 (0)	0 (0)

Nausea/Vomiting	Total	386 (100)	567 (100)
	Grade I	236 (61)	333 (59)
	Grade II	110 (28)	175 (31)
	Grade III	37 (10)	51 (9)
	Grade IV	3 (1)	8 (1)

Diarrhoea	Total	74 (100)	126 (100)
	Grade I	42 (57)	68 (54)
	Grade II	26 (35)	47 (37)
	Grade III	5 (7)	10 (8)
	Grade IV	1 (1)	1 (1)

PPE	Total	379 (100)	2 (100)
	Grade I	195 (51)	2 (100)
	Grade II	120 (32)	0 (0)
	Grade III	62 (16)	0 (0)
	Grade IV	2 (1)	0 (0)

PLD=pegylated liposomal doxorubicin hydrochloride; PPE=palmar-plantar erythrodysesthesia.

(Gordon *et al*, 2001; Smith *et al*, 2002). Then, the costs derived from the treatment of each type of adverse event in each of their severity levels were determined. These results were used to calculate the cost per adverse event and per patient. The estimated cost for each adverse event type was then added to obtain a total adverse event management cost per patient.

### Cost sources. Expert opinion

Costs of study drugs (price ex-factory) and drugs to treat adverse events were taken from the Spanish Catalogue of Medicinal Products 2001. The unit costs of the procedures and tests included in the analysis were collected from the Spanish Data Base of Sanitary Costs 2001 (SOIKOS, 2001) and published literature. Costs were collected in pesetas at 2001 values and converted to euros at the official rate of 166.386 pesetas euro^−1^.

Estimation of resource utilisation associated with treatments of both PLD and T when managing adverse events was made in a retrospective way through an expert panel, which participated for quality assurance and validation purposes. This expert panel consisted of three medical oncologists who usually treat ovarian cancer in representative University Hospitals in Spain and two health economists working in several meetings based on structured questionnaires and discussion. Subsequent meetings between the experts were held to define all resources used in diagnosis and treatment of adverse events.

### Adverse events

The nine adverse events included in this analysis were chosen on the basis of patient perception, frequency and clinical importance, and were the most significant according to the decision of the expert panel based on health-care resource consumption at hospitals. These were the same adverse events evaluated in a previous analysis (Smith *et al*, 2002):

- Anaemia
- Thrombocytopenia
- Neutropenia
- Sepsis
- Fever
- Stomatitis/pharyngitis
- Nausea, vomiting
- Diarrhoea
- PPE: palmar-plantar erythrodysesthesia

Alopecia, one of the most important adverse events occurring in the clinical trial that favoured PLD (Gordon *et al*, 2001), was excluded from the analysis since Spanish hospitals (perspective used) do not treat this adverse event in clinical practice. The panel of experts made an estimation of resources used in the management of adverse events, in terms of the following severity level:

- Mild (Grade I): signs or symptoms were noticed by patients but easily tolerated. The event was not expected to have any influence on the patient's health or well-being, and it had little clinical significance.
- Moderate (Grade 2): patient's usual activities were altered because of the event. It was of some concern to the patient's health or well-being, and may have required medical intervention and/or close follow-up.
- Severe (Grade 3): the adverse event interfered considerably with the patient's usual activities and was of definite concern to the patient and/or posed substantial risk to the patient's health or well-being. The event was likely to require medical intervention and/or close follow-up and may have been incapacitating or life threatening.
- Life threatening/fatal (Grade 4): the patient was incapacitated. The event constituted a high risk to the patient's immediate health or well-being.

## RESULTS

### Base-case

Costs in euros used in the pharmacoeconomic analysis are shown in Table 3

**Table 3:** Unitary costs (euros)

	**PLD**	**Topotecan**
Cost per mg	19.70	63.91
Antiemetic drugs	NR	22.02
Outpatient visit	115.72	
Outpatient consultation	61.32	

PLD=pegylated liposomal doxorubicin hydrochloride; NR=not required.

including cost of drug per mg, cost of the antiemetic drugs required for T, cost of an outpatient visit and cost of an outpatient consultation. Table 4

**Table 4:** Cost estimation of management of each adverse event (euros)

**Adverse event type**	**Grade**	**Costs**
Anaemia	Grade I	0
	Grade II	727.42
	Grade III	326.29
	Grade IV	542.89

Thrombocytopenia	Grade I	0
	Grade II	35.35
	Grade III	194.17
	Grade IV	566.97

Neutropenia	Grade I	0
	Grade II	0.40
	Grade III	149.63
	Grade IV	409.53

Sepsis	Grade I	0
	Grade II	2897.52
	Grade III	2897.52
	Grade IV	2897.52

Fever	Grade I	0
	Grade II	131.84
	Grade III	142.03
	Grade IV	797.26

Stomatitis/pharyngitis	Grade I	0
	Grade II	106.90
	Grade III	724.45
	Grade IV	1330.53

Nausea/vomiting	Grade I	0.02
	Grade II	0.43
	Grade III	356.61
	Grade IV	1118.9

Diarrhoea	Grade I	0
	Grade II	51.19
	Grade III	484.22
	Grade IV	1064.01

PPE	Grade I	0
	Grade II	46.11
	Grade III	168.36
	Grade IV	1036.00

PPE=palmar-plantar erythrodysesthesia.

shows the estimation of costs made by the expert panel when treating adverse events, which increases as the severity level increases. Only anaemia has a grade II severity level more costly to manage than the other grades due to the use of erythropoietin, whereas grades III and IV were considered to be treated with transfusions, which are less costly than erythropoietin.

The final results are calculated in Table 5

**Table 5:** Costs of drug, administration and management of adverse events per patient (euros). Final results of the economic analysis

	**PLD**	**Topotecan**	**Difference**
Total cost: drug+administration	8647.70	8519.94	127.76
Drug cost	7785.43	4256.66	
Administration cost	862.27	4263.29	

Total cost adverse events	967.02	3304.75	−2337.73

Anaemia	334.25	1687.19	
Thrombocytopenia	4.51	337.51	
Neutropenia	45.32	859.82	
Sepsis	48.49	209.61	
Fever	15.53	36.41	
Stomatitis/pharyngitis	163.94	23.00	
Nausea/vomiting	69.48	115.84	
Diarrhoea	210.01	35.37	
PPE	75.49	0.00	

Total cost	9614.72	11 824.69	−2209.97

PLD=pegylated liposomal doxorubicin hydrochloride; PPE=palmar-plantar erythrodysesthesia.

. Costs of the study drug, drug administration, and management of each type of adverse event were added to obtain a total cost per patient for both arms of the study. These results show that the total cost of drug plus administration does not differ substantially between PLD and T, despite the cost of PLD being much higher. This is due to the more frequent and thus more expensive administration necessary with T. However, total medical costs are lower with PLD (9614.72 *vs* 11 824.69 euros), due to the fact that those adverse events that are more frequent with T are more costly to manage, and add significantly to the overall cost.

### Sensitivity analysis

The uncertainty derived from the premises assumed in the study need to be reviewed through a sensitivity analysis to confirm the robustness of our results. Consequently, an analysis was conducted to identify the impact that changes in the most significant variables would have on the study conclusions.

- Increasing/reducing by 50% the length of hospitalisation and the number of outpatient visits when managing adverse events: these parameters were chosen because they are the most influential in the cost of treating the most common adverse events from PLD and T. To favour T, a 50% increase was applied in the case of managing stomatitis/pharyngitis, PPE and diarrhoea (the most important adverse events, in terms of estimated costs values, in treatment with PLD), and the cost of the rest of the adverse events was reduced by 50% (more common in treatment with T). Increasing costs of adverse events common with PLD and reducing costs of adverse events common with T gave results still favourable to PLD, with a cost saving of 1442.85 euros instead of the 2209.97 euros cost saving in the base-case.
- Equalising the number of total cycles administered with the two drugs to 1164 cycles, corresponding to the total cycles in the PLD arm: as T had a more frequent (and thus more expensive) administration, results are in its favour, assuming equal efficacy for the two drugs. Results obtained are still favourable to PLD, with a cost saving of 1625.39 euros.
- Assuming that the antiemetic drugs required to be administered concomitantly with topotecan have a zero cost (the favourable effect of reducing nausea and vomiting is retained but no cost is incurred), another sensitivity analysis was carried out. The results show no difference in final outcomes with a cost saving of 1586.16 euros favouring the treatment with PLD.
- Assuming application of all the above independent changes that favour T: changing hospitalisation length and number of outpatient visits (related to management of adverse events), equalising the number of total cycles required for PLD and T and considering that the concomitant antiemetic drugs used with T have a zero cost (related to the cost of study drugs and their administration). This analysis gave a result which is still favourable to PLD, with a cost saving of 319.99 euros.
- Using lower hypothetical doses for PLD and T. In special populations, the expert panel recommended a dose of 40 mg m^−2^ every 28 days for PLD and 1.25 mg m^−2^ day^−1^ for five consecutive days every 21 days for T. Assuming that equal efficacy still applies to these two lower dose regimens, this change does not influence the study results with a cost saving of 3057.62 euros using PLD.

Table 6

**Table 6:** Sensitivity analysis: total medical costs per patient (euros)

	**PLD**	**Topotecan**	**Difference**
(a) *Changing length of hospitalisation and number of visits*			
Total cost drug+administration	8647.70	8519.94	127.76
Total cost adverse events	1087.04	2657.65	−1570.61

Total cost	9734.74	11 177.59	−1442.85

*(b) Equalising number of cycles*			
Total cost drug+administration	8647.70	7935.36	712.34
Total cost adverse events	967.02	3304.75	−2337.73

Total cost	9614.72	11 240.11	−1625.39

*(c) Cost of antiemetics required for topotecan=0*			
Total cost drug+administration	8647.70	7896.13	751.57
Total cost adverse events	967.02	3304.75	−2337.73

Total cost	9614.72	11 200.88	−1586.16

*(d) Application of a, b and c (above)*			
Total cost drug+administration	8647.70	7397.08	1250.62
Total cost adverse events	1087.04	2657.65	−1570.61
Total cost	9734.74	10 054.73	−319.99

*(e) Lower doses of PLD and topotecan*			
Total cost drug+administration	7090.61	7810.50	−719.89
Total cost adverse events	967.02	3304.75	−2337.73
Total cost	8057.63	11 115.25	−3057.62

PLD=pegylated liposomal doxorubicin hydrochloride.

shows results from the five sensitivity analyses.

## DISCUSSION

Ovarian cancer is the leading cause of cancer deaths of the female reproductive system. The complex therapy used and the increasing number of patients make ovarian cancer a potentially expensive disease to treat, increasing concern to find the most efficient therapy (Berger *et al*, 1998).

This cost-minimisation study has examined the total costs associated with treatment with PLD and T in patients with recurrent epithelial ovarian cancer who failed a first-line platinum-containing regimen. This analysis reveals that total costs of drug and administration per patient do not differ substantially between the two drugs. However, the total medical costs are lower for PLD, due to the additional costs associated with management of adverse events required during treatment with T. Sensitivity analysis showed that the outcomes of our analysis were robust, since changes to variables that favour T were made without changing the results substantially. Even when considering all the assumptions that favour topotecan and bringing them together in the same analysis, the strategy with PLD has a lower cost.

Some limitations of the study must be considered when evaluating the results obtained. Data on which we have based our analysis came from an efficacy study, with an absence of effectiveness data (data in real clinical practice). In addition, resource utilisation was determined using expert opinion. The ideal design for the assessment of medical resource utilisation in this cost-minimisation analysis would have been an observational design, with a prospective economic appraisal to determine resource utilisation; however, time constraints did not allow the execution of this type of study.

Although lower doses might be used in special cases, frequency or severity of adverse events could change (Smith *et al*, 2002). Despite considering lower doses of both drugs according to the expert panel recommendation and assuming equivalent efficacy (not clearly supported by bibliography), sensitivity analysis results remain unaffected and verify the robustness of the model. The impact of direct nonmedical costs and indirect costs were not included, dealing only with direct medical costs. Broader issues of outcomes such as productivity loss or intangible benefits may show differences between the two drugs that were not captured in our analysis. Thus, normally asymptomatic neutropenia does not represent a loss in productivity, but there might be lost time from work due to PPE or stomatitis with PLD. However, if the costs of time-off-work and patient-related costs of travel and waiting time are considered, the analysis would favour PLD, as it is administered once monthly instead of the 5-day cycle every 3 week T treatment.

Quality of life and patients preferences have not been considered in our analysis, and these are important measures in the cancer field, where quantity and quality of life has a high importance. Incorporation of these parameters may affect the results, especially since both administration and incidence of adverse events differ between the two drugs. A cost–utility analysis would have been more interesting, associating costs and quality of life derived from each therapy, and giving patient preferences for each of the two drugs (Earle *et al*, 2000).

Adverse events have been discussed by experts giving the Spanish point of view in usual clinical practice, excluding some events reported in the clinical trial such as alopecia (with a greater percentage in the topotecan group) because it is not routinely treated at hospitals. Adverse event management is different between countries, in terms of choice of drugs, basic treatment pathway, rates of consultations, etc. The analysis cannot be extrapolated to other countries because of these differences.

In summary, on the basis that PLD has overall comparable efficacy with topotecan, a cost-minimisation analysis was performed arriving at the conclusion that PLD is an efficient choice and can be used to reduce costs of treatment in patients with recurrent epithelial ovarian cancer who failed a first-line platinum-containing regimen.
